# Retraction: Comparing long-term outcomes following radical and partial nephrectomy for cT1 renal cell carcinoma in young and healthy individuals

**DOI:** 10.1093/jncics/pkaa056

**Published:** 2020-10-17

**Retraction: Comparing long-term outcomes following radical and partial nephrectomy for cT1 renal cell carcinoma in young and healthy individuals [*JNCI Cancer Spectrum*, 2019: 3(1)]**

Wei Shen Tan, Sebastian Berg, Alexander P Cole, Marieke Krimphove, Maya Marchese, Stuart R Lipsitz, Junaid Nabi, Jesse D Sammon, Toni K Choueiri, Adam S Kibel, Maxine Sun, Steven Chang, Quoc-Dien Trinh

https://doi.org/10.1093/jncics/pkz003

To the Editors: We identified an error in the analysis of our paper, “Comparing long-term outcomes following radical and partial nephrectomy for cT1 renal cell carcinoma in young and healthy individuals” published in *JNCI Cancer Spectrum* (1), while performing a quality control check of previous work done in the lab. Specifically, we utilized RX_SUMM_SURG_PRIM_SITE to determine radical and partial nephrectomy where RX_SUMM_SURG_PRIM_SITE=50 was radical nephrectomy and mistakenly used RX_SUMM_SURG_PRIM_SITE=40 for partial nephrectomy. We ran the analysis using the correct code for partial nephrectomy (RX_SUMM_SURG_PRIM_SITE=30). Following inverse probability of treatment weighting to adjust for confounding factors, we found a hazard ratio of 0.59 (95% confidence interval = 0.46 to 0.76, *P*<.001) suggesting an overall survival difference favoring partial nephrectomy. This finding is in contrast to our previous finding reported in the published article (hazard ratio = 0.83, 95% confidence interval = 0.63 to 1.10, *P*=.196). Two data analysts have examined the code and were able to reproduce the updated results independently. This changes not only the results but also the conclusions of the article as there was an overall difference in overall survival favoring partial nepthrectomy and therefore this patient cohort may not have sufficient renal reserve over their lifetime, and preserving nephrons by partial nephrectomy may be necessary.

Because of this honest error, we have decided to retract the above article. We apologize for this oversight and error in coding.
